# Efficacy of low dose of rituximab to treat systemic lupus erythematosus

**DOI:** 10.3389/fimmu.2026.1806879

**Published:** 2026-06-16

**Authors:** Yan Chen, Limei Xu, Yanqing Wang, Xuwei Yang, Wenzhong Que

**Affiliations:** 1 Department of Rheumatology, Fuzhou First General Hospital Affiliated with Fujian Medical University, Fuzhou, China; 2 Department of Rheumatology, Fujian Medical University Union Hospital, Fuzhou, China

**Keywords:** belimumab, efficacy, low dose, retrospective analysis, rituximab, systemic lupus erythematosus

## Abstract

**Objective:**

This study aimed to compare the efficacy and safety of low-dose rituximab (RTX, 100 mg/month) versus belimumab (BEL, 10 mg/kg), as a control, in the treatment of systemic lupus erythematosus (SLE).

**Methods:**

A retrospective analysis was conducted on 117 SLE patients who received low-dose RTX (n=51) or BEL (n=66) at Fujian Medical University Union Hospital between January 2020 and June 2023. Data on baseline characteristics, laboratory parameters (platelet count, creatinine, proteinuria, complement C3/C4, erythrocyte sedimentation rate (ESR)), disease activity (SLEDAI−2K), glucocorticoid dosage, concomitant immunosuppressant use, efficacy assessments, and adverse events were collected. Paired t-tests or Wilcoxon tests were used for within-group comparisons, and independent-sample t-tests or Mann-Whitney U tests for between-group comparisons.

**Results:**

No significant differences were observed between the two groups at baseline for SLEDAI-2K, glucocorticoid levels, or anti-dsDNA (*p* > 0.05). After treatment, both groups showed significant improvements in SLEDAI-2K, dsDNA levels, and glucocorticoid dosage (all *p* < 0.05), with a greater reduction in glucocorticoid dosage in the BEL group (*p* = 0.02). Both treatments increased complement C3/C4 and decreased immunoglobulins (*p* < 0.05), with no significant between-group differences. Low-dose RTX achieved a overall efficacy of 39.2% versus 57.6% for BEL. Both agents increased platelet count, improved hypoalbuminemia and hypercoagulability, and decreased ESR; BEL was superior in reducing D-dimer (*p* = 0.03). In renal outcomes, both reduced proteinuria equivalently; BEL improved creatinine while RTX caused a small but clinically acceptable rise in creatinine. Adverse events occurred in 12.12% (BEL) and 7.8% (RTX) of patients, respectively (*p* > 0.05), with no unexpected safety signals. Concomitant immunosuppressant use also varied between groups, with mycophenolate mofetil (MMF) more common in BEL and Cyclosporine A (CsA) in RTX.

**Conclusion:**

Low-dose RTX (100 mg/month) can be regarded as a treatment option for SLE, based on its comparable efficacy to BEL in improving disease activity, increasing platelet count, and ameliorating proteinuria. Although BEL shows greater advantages in reducing glucocorticoid dosage and improving hypercoagulability, the overall evidence supports low-dose RTX as an effective and safe alternative for SLE, with a favourable safety profile.

## Introduction

Systemic lupus erythematosus (SLE) is a systemic autoimmune disease characterized by the formation of autoantibodies, deposition of antibodies in tissues, and activation of the complement system, ultimately leading to end-organ damage and dysfunction (1, 2). Clinical intervention for SLE primarily relies on glucocorticoids, immunosuppressants, and other therapeutic regimens (3). However, SLE predominantly affects women of childbearing age, and effective drugs such as cyclophosphamide may pose substantial short- and long-term risks to fertility, teratogenicity, and carcinogenicity (4). Despite an incomplete understanding of the pathogenesis of SLE, the interplay of genes, environmental factors, and innate and adaptive immune responses significantly contributes to the immunological alterations observed in SLE patients (2). Substantial evidence underscores the pivotal role of B cells in the pathogenesis of SLE. Consequently, biologics such as rituximab (RTX) and belimumab (BEL) have emerged as key players in SLE treatment (3, 5–8).

The primary mechanism of BEL is to inhibit B-cell activation and autoantibody production, which made it the first FDA-approved biologic for SLE (9, 10). Although clinical trials and real-world use have confirmed its ability to reduce disease activity and glucocorticoid dependence, some patients still have a suboptimal response (11–13). Regarding safety, the Phase 4 BASE study noted a low overall adverse event rate but reported an imbalance of serious infusion/hypersensitivity reactions, depression, and suicidality compared to placebo, whereas on-treatment mortality remained similar between groups. In contrast, a real-world study from China found that BEL was generally well tolerated in adult and paediatric SLE patients, with no new safety signals identified, although longer-term follow-up beyond 24 weeks is still needed to strengthen this evidence (14).

RTX is the first chimeric human-mouse anti-CD20 monoclonal antibody to be applied in clinical practice. RTX functions by binding to the CD20 antigen on B lymphocytes and inducing B cell lysis through complement-dependent cytotoxicity and antibody-dependent cell-mediated cytotoxicity. Additionally, RTX can inhibit B lymphocyte proliferation and induce B cell apoptosis (15). Based on 2020 Chinese guidelines for the diagnosis and treatment of SLE, RTX has been proposed for the application in patients with refractory lupus nephritis (LN) and hematological involvement, owing to its superiority in promoting disease control and reducing glucocorticoids usage (16). RTX can also be used as a second-line treatment for relapsing/refractory SLE, especially in patients who have failed CYC-based regimens, and for severe autoimmune thrombocytopenia (3, 17).

In SLE, both thrombocytopenia and LN are driven by anti-dsDNA autoantibodies and immune complex deposition, both mediated by B cells and plasma cells. Initially, RTX was used in SLE patients at doses equivalent to those for lymphoma (375 mg/m^2^ weekly for 4 weeks). Although this regimen showed good efficacy, it also carried an increased risk of spontaneous infection due to reduced immunoglobulin levels (18). Notably, a mechanistic clue emerged from RTX treatment of SLE-associated thrombocytopenia: low-dose RTX regimens (100 mg/week for 4 weeks, or two infusions of 200mg every 2 weeks) achieved B-cell depletion comparable to standard-dose regimens (19, 20). Given the shared pathogenic mechanisms between thrombocytopenia and LN, this finding suggests that such low-dose regimens might also induce renal improvement in LN.Thus, a regimen validated in thrombocytopenia may logically be beneficial for SLE and even LN.

Despite its widespread use in B-cell malignancies and autoimmune diseases, the standard RTX dose (375 mg/m^2^ weekly for 4 weeks) is limited in SLE by infectious concerns and high cost. Consequently, recent studies have explored ultra-low-dose regimens. In LN patients, an ultra-low-dose RTX regimen (100 mg weekly for 4 weeks, followed by 100 mg every 2-3 months) achieved a 12-month renal remission rate of up to 94.1% and a complete B-cell depletion rate of 97.1% after the initial 4 weeks, with no serious adverse events (21). Another reduced-dose study (375 mg/m² ×1, then 100 mg every 3 months) reported a cumulative renal remission rate of 87% among 23 refractory/relapsed LN patients (22). Based on these encouraging results, the present study aimed to evaluate an even lower-dose RTX regimen (100 mg per month) in the treatment of SLE, using BEL as the control. We performed a comprehensive review of the data from hospitalized SLE patients receiving either low-dose RTX or BEL, and proposed an optimal treatment option for SLE.

## Materials and methods

### Patients

This retrospective study was approved by the Ethics Committee (No. 2023KY204) on November 8, 2023, with a waiver of informed consent. This study reviewed medical records without patient interaction, posed no additional risks or costs, and strictly protected patient privacy. Clinical data and laboratory test results of 117 SLE patients admitted to our hospital from January 2020 to June 2023 were analyzed, with follow-up until June 2023 (median follow-up duration: 5.9 months; range: 0.5 - 34.9 months). Among these patients, 66 received BEL treatment (6 males, 60 females; mean age 30.98 ± 10.46 years, disease duration 5 (2, 10) years, number of administratons 7 (4, 8). The other 51 patients received RTX treatment (1 male, 50 females, mean age 34.67 ± 12.08 years, disease duration 4 (2, 8) years, number of administratons 2 (2, 4). All efficacy outcomes were assessed at the last available follow-up (June 2023), with baseline values collected before treatment initiation (**Table 1**).

**Table 1:** General data of two groups.

Indicators	BEL (N = 66)	RTX (N = 51)
Age (years)	30.98 ± 10.46	34.67 ± 12.08
Gender (female)	60 (90.9%)	50 (98%)
Disease duration (years)	5 (2, 10)	4 (2, 8)
Treatment duration (days)	221 (98, 285)	76 (48.5, 292)
Number of administrations of (BEL/RTX)	7 (4, 8)	2 (2, 4)
HGB (g/L)	109.0 ± 20.9	112.9 ± 23.6
Platelet count (×10^9^/L)	202.55 ± 97.81	169.88 ± 109.70
Renal involvement n (%)	48 (72.7%)	28 (54.9%)
Creatinine (umol/L)	51 (45, 61)	54.5 (42.75, 65.25)
SLEDAI-2000 score	9 (8, 12)	8 (4.5, 12.5)

Inclusion criteria: 1. Conformity to the EULAR/ACR 2019 classification criteria for SLE (23). 2. There was no abnormalities in heart, lung, and liver function.

Exclusion criteria: 1. Inability to assess efficacy or unknown efficacy. 2. Presence of severe neurological disease. 3. Lactation or pregnancy.

Treatment options included background therapy, including glucocorticoids, antimalarials, immunoagents, or cytotoxic agents, administered in conjunction with BEL or RTX. The administration involved 10 mg/kg intravenous infusion of BEL (according to the prescribing information: the first three infusions every two weeks, followed by every 4 weeks thereafter) and 100 mg /month intravenous infusion of RTX.

### Data collection

Comprehensive demographic data, including gender, age, weight, and clinical particulars such as disease duration, target organ involvement, and SLEDAI-2K scores, were collected. Laboratory analyses included routine blood tests, urinalysis, liver and kidney function tests, dsDNA levels, serum complement levels, quantitative immunoglobulin levels, and erythrocyte sedimentation rate (ESR). Treatment details, such as pre- and post-treatment glucocorticoid dosages, number of administrations, treatmen durations and adverse events, were also documented. The collected data were analyzed to compare differences in SLEDAI-2K scores, glucocorticoid dosage, dsDNA levels, platelet (PLT) counts, immunoglobulin and complement levels, ESR, serum albumin, lactate dehydrogenase (LDH), D-dimer, creatinine, urea nitrogen, and urinary protein before and after treatment with BEL or RTX. Specifically, glucocorticoid dosages were quantified as prednisolone-equivalent doses based on standard conversion (5 mg prednisone = 4 mg methylprednisolone). The SLEDAI-2K score encompasses 24 clinical metrics spanning nine organ systems, with scores ranging from 0 to 105. Disease activity was classified as essentially no activity (≤ 4), mild activity (5-9), moderate activity (10-14), and severe activity (≥ 15). Adverse events (AEs) were defined and graded according to the National Cancer Institute Common Terminology Criteria for Adverse Events (CTCAE), version 5.0 (24).

### Evaluation of therapeutic efficacy

The 2021 DORIS definition of remission in SLE (25): (1) Clinical SLEDAI = 0. (2) Physician Global Assessment (PGA) < 0.5 (0-3). (3) Irrespective of serology. (4) The patient may be on antimalarials, low-dose glucocorticoids (prednisolone ≤ 5 mg/day), and/or stable immunosuppressives including biologics. If DORIS alleviation cannot be achieved, then LLDAS is recommended as the target (26): (1) SLEDAI-2000 score is ≤ 4; (2) PGA score is ≤ 1; (3) The treatment plan may include antimalarial drugs (such as hydroxychloroquine), low-dose glucocorticoids (prednisone ≤ 7.5 mg/day), and / or stable doses of immunosuppressants / biological agents.

1. Complete remission (CR): clinical SLEDAI = 0, PGA < 0.5, prednisone ≤ 5 mg/day (or equivalent), and normal anti-dsDNA (if applicable), consistent with the DORIS (Definitions of Remission in SLE) criteria.
2. Partial remission (PR): achievement of Lupus Low Disease Activity State (LLDAS), i.e., SLEDAI-2K ≤ 4, PGA ≤ 1, prednisone ≤ 7.5 mg/day, and no new organ involvement.
3. Ineffectiveness: not meeting the criteria for either CR or PR.

The overall efficacy was calculated as follows: Overall efficacy = (Total number of cases − Number of ineffective cases) / Total number of cases × 100.00%.

### Statistical analysis

Statistical analysis was conducted using SPSS 26.0 software. Descriptive data for normally distributed variables were presented as mean ± standard deviation (SD), while non-normally distributed variables were presented as median or range. Count data were expressed as [n (%)]. Changes before and after treatment were compared using paired-sample t-tests or paired Wilcoxon signed-rank tests, based on the normality of the data. Differences between the two groups were assessed using independent-sample t-tests or Mann-Whitney U tests. Statistical significance was set at *p*  < 0.05 for all tests. As an exploratory retrospective study, no multiple-testing correction was applied; *p*-values are nominal and require prospective validation.

## Results

### Evaluation of overall efficacy

Despite lower efficacy than BEL (39.2% vs. 57.6%), RTX still showed a 39.2% response rate, confirming its partial effect and justifying continued exploration (**Table 2**).

**Table 2:** Comparison of overall efficacy of BEL and RTX.

Group	Complete remission	Partial remission	Ineffectiveness	Overall efficacy
BEL (N = 66)	19 (28.8%)	19 (28.8%)	28 (42.4%)	38 (57.6%)
RTX (N = 51)	15 (29.4%)	5 (9.8%)	31 (60.8%)	20 (39.2%)
χ^2^				6.981
*p*				0.0305

### Comparison of SLEDAI-2K, glucocorticoids levels, and dsDNA levels before and after treatment between the two groups

There was no significant difference in SLEDAI-2K, glucocorticoid levels, and dsDNA levels between the two groups before the treatment (*p* > 0.05). After treatment, SLEDAI-2K, dsDNA levels and glucocorticoid dosage exhibited a notable decrease (both *p*  < 0.05), and the reduction in glucocorticoid dosage in the BEL group was significantly greater than in RTX group (*p*  = 0.02, **Table 3**). Both treatments ameliorated the overall condition of patients and reduced glucocorticoid dosage.

**Table 3:** Comparison of SLEDAI-2K, glucocorticoid levels, and dsDNA levels before and after treatment in two groups.

Group	SLEDAI-2K score		Dosage of glucocorticoids (mg)		dsDNA (IU/ml)	
	Before	After	Before	After	Before	After
BEL	9.74 ± 4.65	4.59 ± 3.49	20 (10, 35)	5 (5, 10)	44.4 (13.75, 100)	21.68 (6.02, 86.8)
RTX	9.41 ± 5.84	5.22 ± 4.61	30 (10, 50)	10 (5, 30)	22.84 (12.54, 100)	11.9 (4.5, 47.1)
t/z	0.341	-0.835	-1.84	-2.29	-1.12	-1.70
*p*	0.094	0.088	0.066	0.022	0.263	0.088

### Changes of serum complement and immunoglobulin levels in two groups

Serum complement C3 and C4 levels increased in patients treated with BEL and RTX (*p*  < 0.05). Although the increases in C3 and C4 levels were higher in RTX-treated patients than in BEL-treated patients, the differences were not significant (C3, t = -1.74, *p* = 0.53; C4, t = -1.23, *p*  = 0.82). Immunoglobulins (IgG, IgA, and IgM) decreased after treatment compared with baseline levels (*p* < 0.05), but there were no significant differences between the RTX and BEL groups (*p* > 0.05; **Table 4**).

**Table 4:** Changes of serum complement and immunoglobulin levels in two groups.

Group	C3 (g/L)		C4 (g/L)		IgG (g/L)		IgA (g/L)		IgM (g/L)	
	Before	After	Before	After	Before	After	Before	After	Before	After
BEL	0.61± 0.21	0.69± 0.21	0.13± 0.07	0.15± 0.07	10.6 (7.71, 14.8)	10.4 (8.27, 13)	2.25 (1.63, 2.88)	1.92 (1.38, 2.41)	0.83 (0.49, 1.18)	0.70 (0.45, 0.84)
RTX	0.63± 0.22	0.75± 0.20	0.13± 0.07	0.17± 0.07	11.9 (8.01, 16.95)	10.3 (7.55, 14.3)	2.14 (1.46, 2.74)	1.95 (1.32, 2.67)	0.87 (0.59, 1.18)	0.69 (0.41, 0.87)
t/z	-0.56	-1.74	-0.23	-1.23	-1.14	-0.24	-0.12	-0.01	-0.75	-0.51
*p*	0.54	0.53	0.29	0.82	0.25	0.81	0.91	0.99	0.46	0.61

### Changes in general biochemical indices in two groups

There was no significant difference in general biochemical indices between the two groups before treatment (*p* > 0.05). In further descriptive detail, the proportion of patients with baseline thrombocytopenia (PLT count <100 × 10^9^/L) was 15.2% (10/66) in the BEL group and 29.4% (15/51) in the RTX group. Following administration of BEL and RTX, patients showed decreased ESR and D-dimer levels (*p*  < 0.05), along with increased serum albumin and PLT levels (*p*  < 0.05). Although D-dimer levels were lower after BEL treatment than after RTX (*p*  = 0.03), suggesting that BEL had a superior effect on D-dimer reduction, RTX also significanltly inproved the hypercoagulable state (**Table 5**).

**Table 5:** Changes in general biochemical indices in two groups.

Group	ESR (g/L)		Serum albumin (g/L)		PLT (×10^9^/L)		D-dimer (μg/L)	
	Before	After	Before	After	Before	After	Before	After
BEL	21 (11, 48)	12 (6, 22)	34.47 ± 6.35	36.39± 5.83	202.55 ± 97.81	224.76 ± 69.99	0.36 (0.22, 0.79)	0.24 (0.15, 0.35)
RTX	19 (8, 61.75)	12 (7, 17)	34.36 ± 7.83	37.49± 6.30	169.88 ± 109.70	200.08 ± 96.71	0.45 (0.27, 0.92)	0.32 (0.21, 0.73)
t/z	-0.20	-0.29	-0.43	-0.93	1.70	1.60	-0.87	-2.20
*p*	0.84	0.77	0.33	0.33	0.32	0.09	0.38	0.03

### Changes in renal function and urinary protein in two groups

At baseline, there were no significant differences in urine protein grades or serum creatinine levels between the BEL and RTX groups, confirming comparability. Both treatments significantly reduced urine protein grades from baseline (both *p* < 0.01), and post-treatment intergroup comparison showed no significant difference in the distribution of urine protein grades (χ² = 7.123, *p* = 0.129, **Table 6**), indicating equivalent efficacy in improving proteinuria. Regarding other renal function parameters, the BEL group showed a significant decrease in creatinine (z = −2.23, *p* = 0.027) and LDH (*p*  = 0.003), while the RTX group showed a significant increase in creatinine (z =2.69, *p*  = 0.007) (**Table 7**). The percent changes from baseline for creatinine, urea nitrogen, and LDH were significantly different between the two groups (*p*  = 0.002, *p*  = 0.003, and *p*  = 0.001, respectively). Nevertheless, all post−treatment creatinine values remained within the normal range. In summary, BEL showed a trend toward improving renal function parameters, whereas RTX caused a significant but clinically acceptable rise in creatinine. Bboth agents had favorable renal safety profiles and were equally effective in reducing proteinuria.

**Table 6:** Urine dipstick protein grades before and after treatment in two groups.

Group	Urine protein			
	Results	Before	After	*p*-value
BEL (N = 66)	–	20 (30.30%)	29 (43.94%)	*p* = 0.0095
	±	10 (15.15%)	10 (15.15%)	
	+	16 (24.24%)	9 (13.64%)	
	++	11 (16.67%)	14 (21.21%)	
	+++	6 (9.09%)	4 (6.06%)	
	++++	3 (4.55%)	0 (0.0%)	
RTX (N = 51)	–	24 (47.06%)	32 (62.75%)	*p* = 0.0011
	±	7 (13.73%)	4 (7.84%)	
	+	4 (7.84%)	4 (7.84%)	
	++	7 (13.73%)	7 (13.73%)	
	+++	8 (15.69%)	4 (7.84%)	
	++++	1 (1.96%)	0 (0.0%)	
χ²		7.123		
*p*		0.129		

**Table 7:** Changes in renal function in two groups.

Group	Creatinine (μmol/L)		Urea Nitrogen (mmol/L)		LDH (U/L)	
	Before	After	Before	After	Before	After
BEL	51 (45, 61)	48 (41, 57.25)	4.7 (3.5, 6.1)	4.2 (3.5, 5.78)	195.5 (165.25, 240.25)	177 (155, 205.5)
RTX	54.5 (42.75, 65.25)	57 (49.25, 64.75)	5.3 (3.8, 6.1)	5.4 (4.43, 7.2)	200 (166, 233)	229 (185, 292)
t/z	-0.24	-3.1	-1.02	-3.0	-0.23	-3.87
*p*	0.81	0.002	0.31	0.003	0.82	0.001

### Comparison of the incidence of adverse events in two groups

Among the 117 SLE patients in this study, 8 adverse events (12.12%) were observed in the BEL-treated group. Of these, 5 were infectious adverse events, including 3 cases of upper respiratory infection (improved after treatment with oseltamivir) and 2 cases of pneumonia (improved after antibiotic therapy). The other 3 events were transaminitis (transaminase levels returned to normal after liver protection therapy). In the RTX-treated group, a total of 4 adverse events (7.8%) were reported. Among them, 1 was an infectious adverse event (upper respiratory infection, also improved after oseltamivir). Two events were anaphylaxis: one patient developed abdominal and lower limb rashes with pruritus during the last intravenous injection of RTX, which did not recur after administration of dexamethasone (5 mg); the other patient experienced dyspnea and tachycardia during intravenous injection, which improved after dexamethasone treatment. One patient had dizziness, which improved after stopping the infusion and resting for 1 hour, and then the RTX infusion was resumed smoothly. In summary, the incidence of adverse events was lower during RTX group, but the difference between the two groups was not statistically significant (**Table 8**).

**Table 8:** Comparison of the incidence of adverse events in two groups.

Group	Dizziness	Infection	Alanine aminotransferase increased	Anaphylaxis	Incidence
BEL (N = 66)	0 (0.00%)	5 (7.57%)	3 (4.55%)	0 (0.00%)	8 (12.12%)
RTX (N = 51)	1 (1.96%)	1 (1.96%)	0 (0.00%)	2 (3.92%)	4 (7.8%)
χ²					0.572
*p*					0.449

### Distribution of concomitant immunosuppressants

The proportion of patients using any immunosuppressant was 74.2% (49/66) in the BEL group and 84.3% (43/51) in the RTX group. Regarding specific agents (multiple choices allowed), mycophenolate mofetil (MMF) was used more frequently in the BEL group than in the RTX group (39.4% vs. 19.6%), whereas Cyclosporine A (CSA) was markedly more common in the RTX group (47.1% vs. 7.6%). The use of cyclophosphamide (CYC) (12.1% vs. 15.7%) and Tacrolimus (TAC) (10.6% vs. 15.7%) was comparable between the two groups. In terms of monotherapy, MMF alone was the predominant regimen in the BEL group (33.3%), while CSA alone was most frequent in the RTX group (31.4%). Other combination regimens, such as MMF + TAC and MMF + CSA, were also observed in a small subset of patients. Detailed distribution of concomitant immunosuppressants is provided in **Supplementary Table 1**.

## Discussion

In this study, both BEL and low-dose RTX achieved a certain degree of efficacy in the treatment of SLE. After treatment, both groups showed significant improvements in SLEDAI-2K scores, dsDNA levels, and glucocorticoid dosage, with the BEL group demonstrating a greater advantage in steroid reduction. Serum complement C3 and C4 levels increased, while immunoglobulins (IgG, IgA, IgM) decreased similarly in both groups. Regarding general biochemical indices, ESR and D-dimer decreased, while albumin and platelets increased in both arms; BEL was more effective in lowering D-dimer levels. In terms of renal function, BEL reduced serum creatinine, blood urea nitrogen, and LDH, whereas the RTX group showed mild increases in these parameters, although all post-treatment creatinine values remained within the normal range. Adverse events were slightly less frequent in the RTX group, and both treatments demonstrated generally favorable safety profiles.

BEL represents a pioneering advancement as the first biologic agent approved by the FDA for the treatment of SLE (27). Its mechanism involves high-affinity binding to soluble B-lymphocyte stimulator (BLyS), blocking its binding to receptors, thereby inhibiting B-cell proliferation, reducing differentiation into antibody-producing plasma cells, and suppressing autoantibody production (9). The 2020 Chinese Guidelines for the Diagnosis and Treatment of SLE indicate that BEL can improve serological markers, reduce glucocorticoid dependence, and alleviate proteinuria and relapse in patients with LN (28). Multiple large-scale, multicenter, randomized double-blind controlled phase III clinical trials have consistently demonstrated the efficacy of BEL in SLE (29–32), particularly in non-severe active LN, where BEL (with or without MMF) significantly reduced proteinuria and organ damage. Nevertheless, some patients do not respond well to BEL, highlighting the ongoing need to explore novel treatment options.

Mechanistically, RTX directly depletes CD20^+^ B cells by targeting the CD20 antigen, whereas BEL reduces the survival of autoreactive B cells by inhibiting the BLyS signaling pathway (33). Given their complementary actions, strategies combining or sequencing these two agents have garnered increasing attention. RTX has a pronounced effect in severe SLE, especially in refractory thrombocytopenia; however, standard-dose RTX may increase infection risk due to reduced globulin levels. Sequential therapy with RTX followed by BEL can significantly alleviate clinical symptoms, normalize serological indicators, and reduce background immunosuppressive therapy (34). Early studies such as SynBioSe and BEAT-LUPUS suggested that sequential RTX-BEL therapy could reduce autoantibody levels, ameliorate NETosis, and improve disease activity, with a considerable proportion of patients achieving lupus low disease activity state (LLDAS). In LN, combination therapy may have particular value - BEL may delay the reconstitution of autoreactive B cells after RTX, thereby prolonging remission. Nevertheless, the BLISS-BELIEVE and CALIBRATE trials did not confirm superiority of combination therapy over monotherapy, suggesting that further optimization of patient selection strategies is needed (35). In light of these complementary mechanisms, we further examined the renal function changes in our two groups, which showed interesting differences.

Of note, in the present study, the RTX group showed a mild increase in serum creatinine that remained within the normal range, whereas the BEL group exhibited a trend toward improved renal function parameters. Reviewing the literature, RTX still achieves high renal response rates in refractory LN. A single-center cohort study showed that the renal response rate at 3 months after RTX treatment was 64.5%, and at 12 months was 65.3%; proteinuria and SLEDAI-2K scores decreased significantly, and the mean daily prednisone dose was reduced from 11.3 mg to 6.1 mg (*p*  = 0.005) (36). Nevertheless, reversible mild elevations in renal function parameters after RTX treatment are documented in the literature. For example, a comparative analysis found that the treatment discontinuation rate at 12 months was highest in the RTX group (50%), and the corticosteroid dose (median 10 mg/day) was higher than that in the BEL group (7.5 mg/day) (37). These findings suggest that for patients with high baseline creatinine, caution is warranted when choosing RTX therapy.

Furthermore, although overall platelet levels were comparable at baseline, a descriptively higher proportion of patients in the RTX group had baseline thrombocytopenia (< 100 × 10^9^/L) compared with the BEL group. This reflects the real-world, retrospective nature of our study, where treatment selection was based on clinical characteristics rather than random allocation. Consistent with the above, patients with active LN and proteinuria - typically a chronic, indolent course - were more likely to receive BEL, and also received MMF more frequently. In contrast, patients with severe thrombocytopenia requiring rapid haematological improvement were preferentially treated with RTX due to its faster onset, and these patients frequently received CSA as first-line immunosuppressant, consistent with our findings that CSA use was markedly higher in the RTX group (as shown in **Supplementary Table 1**). Consequently, the observed distribution is driven by disease phenotype and urgency, rather than by random allocation.

Regarding safety, in the present study, a low-dose RTX regimen was used, and we observed that the infection risk was similar between the RTX and BEL groups, consistent with the literature reporting only a modest increase in infection risk with low-dose therapy (38). A network meta-analysis has shown that low-dose B-cell-targeted therapy is associated with only a modest increase in infection risk, and no significant increase in serious infection risk has been observed (38). A prospective cohort study from the BILAG-BR registry (n=1048) demonstrated that in patients with active SLE, the risk of serious infection with RTX, BEL, and standard immunosuppressive therapy was similar; key risk factors for infection included multimorbidity, hypogammaglobulinemia, and glucocorticoid dosage exceeding 10 mg/day (18). Although the infection risk of low-dose B-cell-targeted therapy is slightly increased compared with placebo (RR 1.05), it remains generally manageable (38). In terms of overall efficacy, systematic reviews have noted that although clinical trial results for RTX and BEL differ somewhat - BEL met its primary endpoints in several phase III trials, whereas RCTs of RTX failed to demonstrate superiority over placebo - in real-world clinical practice, both agents are effective in SLE (39).

In conclusion, both BEL and low-dose RTX demonstrate favorable efficacy and safety in the treatment of SLE. BEL shows certain advantages in steroid reduction, improvement of D-dimer levels, and renal function parameters, while low-dose RTX is also applicable in general SLE cases with a manageable infection risk. For patients with elevated baseline creatinine, even if mild increases in renal function parameters are observed and remain within the normal range, renal function should be closely monitored when using RTX. Future head-to-head clinical trials with larger sample sizes are needed to validate these findings and to explore biomarkers that can predict the efficacy of these two agents, thereby enabling individualized treatment.

However, this study has several limitations. First, as a single-center retrospective study, it is prone to selection bias, and treatment duration and infusion numbers were unbalanced between groups. Second, the distribution of concomitant immunosuppressants also differed (MMF more in BEL, CSA more in RTX), reflecting real-world clinical decisions rather than randomization. Future studies could employ stratified analyses or propensity score matching to adjust for such imbalances. Third, the sample size was small and follow-up short, longer-term prospective trials with larger sample sizes are needed to validate our findings.

## Conclusion

In conclusion, low-dose RTX (100 mg/month) can be regarded as a treatment option for SLE, based on its meaningful response rate, comparable efficacy to BEL across multiple endpoints, and favourable safety profile; despite some differences in glucocorticoid reduction, D-dimer, and creatinine, the overall evidence supports low-dose RTX as an effective and safe alternative for SLE.

## Data Availability

The original contributions presented in the study are included in the article/**Supplementary Material**. Further inquiries can be directed to the corresponding authors.

## Supplementary material

Supplementary Table 1 Distribution of Concomitant Immunosuppressants in two groups.
