# Supplementary material for: Efficacy of low dose of rituximab to treat systemic lupus erythematosus
Source: Front Immunol. 2026 Jun 16;17:1806879. doi: 10.3389/fimmu.2026.1806879 (PMC13314405; doi:10.3389/fimmu.2026.1806879)
Supplement: Supplementary Table 1 — Distribution of Concomitant Immunosuppressants in two groups. [file Table1.pdf]

Supplementary Table 1. Distribution of Concomitant Immunosuppressants in two groups.

Group	BEL (N = 66)	RTX (N = 51)
Any immunosuppressant use, n (%)	49 (74.2%)	43 (84.3%)
No immunosuppressant use, n (%)	17 (25.8%)	8 (15.7%)
Specific agents (multiple choices allowed)*		
Mycophenolate mofetil (MMF)	26 (39.4%)	10 (19.6%)
Cyclophosphamide (CYC)	8 (12.1%)	8 (15.7%)
Tacrolimus (TAC)	7 (10.6%)	8 (15.7%)
Cyclosporine A (CSA)	5 (7.6%)	24 (47.1%)
Combination regimens		
MMF alone	22 (33.33%)	2 (3.9%)
CYC alone	6 (9.1%)	5 (9.8%)
TAC alone	5 (7.6%)	5 (9.8%)
CSA alone	5 (7.6%)	16 (31.4%)
MMF + CYC	1 (1.5%)	0 (0.0%)
MMF + TAC	2 (3.0%)	4 (7.8%)
MMF + CSA	0 (0.0%)	3 (5.9%)
CYC + CSA	0 (0.0%)	2 (3.9%)
Other combinations or drugs	8 (12.1%)	6 (11.8%)

*Note: Percentages in the "Specific agents" section refer to the proportion of patients in each group who used the respective drug (regardless of concomitant use of other immunosuppressants). Percentages may add up to >100% due to multiple choices.
